# Potential new role of the GHSR-1a-mediated signaling pathway in cardiac remodeling after myocardial infarction (Review)

**DOI:** 10.3892/ol.2014.2245

**Published:** 2014-06-12

**Authors:** MING-JIE YUAN, HE HUANG, CONG-XIN HUANG

**Affiliations:** Department of Cardiology, Renmin Hospital of Wuhan University, Wuhan, Hubei 430060, P.R. China

**Keywords:** ghrelin, GHSR-1a, myocardial infarction, cardiac remodeling

## Abstract

The gastrointestinal hormone ghrelin has important cardiovascular protective effects, however, its specific mechanisms are not yet completely understood. Recent studies have shown that the ghrelin receptor, growth hormone secretagogue receptor type 1a (GHSR-1a), regulates cell proliferation, apoptosis and inflammation-related signaling pathways. In human aortic endothelial cells, ghrelin activates NO production through AMP-activated protein kinase (AMPK) and Akt activation, and these effects can be blocked by knockdown of GHSR-1a. Obese mice have been found to exhibit an increased GHSR-1a content and expression in the heart, associated with an increase in phosphatidylinositol 3-kinase (PI3K) content and an increase AKT content and phosphorylation. Furthermore, GHSR-1a expression was observed to be increased in heart failure after myocardial infarction (MI) in rats. Given such complexity in GHSR-1a signaling and crosstalk with the AMPK and PI3K/Akt signaling pathways, both of which are well-known factors involved in cardiac remodeling after MI, we speculate that GHSR-1a signaling may play a regulatory role in cardiac protection and hope to identify new drugs targets. However, to date, no direct association between GHSR-1a and cardiac remodeling has been found. Therefore, further studies are required.

## 1. Introduction

Ventricular remodeling after myocardial infarction (MI) is defined as progressive chamber dilation and wall thinning, which lead to heart failure. Remodeling is mediated by active processes of inflammation, fibrosis and cardiomyocyte drop-out over the weeks and months following infarction (1). Therefore, avoiding the damage caused by ischemia and protecting the structure and function of cardiac remodeling have always remained a focus of basic and clinical research (2). To the best of our knowledge, ghrelin appears to have potential as a therapeutic target for cardiovascular diseases (3).

Ghrelin, a peptide of 28 amino acids, was first reported by Kojima *et al* in rat and human stomachs, in 1999 (4). As the endogenous ligand of the growth hormone secretagogue receptor, ghrelin was initially identified to be a strong stimulant for the release of growth hormone (GH). Subsequently, ghrelin and its various receptors were found to be ubiquitous in numerous organ and tissue types. Moreover, they have been demonstrated to participate in the regulation of cardiovascular (5), pulmonary and immune functions (6), as well as cell proliferation and apoptosis (7,8). Furthermore, we previously identified that ghrelin is capable of inhibiting cardiac remodeling and improvements in cardiac function following MI, through an anti-inflammation effect (9). Therefore, these findings suggest that targeting ghrelin may be a potential therapy for cardiovascular diseases. However, until now, its specific mechanisms have not been completely understood.

## 2. Ghrelin/GHSR-1a-mediated signaling pathway

Apart from the ability to stimulate GH secretion and to exert regulatory effects on appetite and metabolism, it has become increasingly evident that ghrelin has a number of effects on the cardiovascular system (10) via a GH-independent pathway, such as reducing myocardial oxygen consumption, anti-inflammatory action (9), inducing anti-myocardial apoptosis, inhibiting myocardial fibrosis (11) and suppressing the sympathetic nervous system (12,13).

The growth hormone secretagogue receptor type 1a (GHSR-1a) has been found to be distributed in vascular endothelium, myocardium and monocytes (14–16), and cultured cardiomyocytes were found to be able to synthesize ghrelin (17), suggesting that ghrelin may exert a paracine/autocrine function in cardiac muscle. Cellular signals carried by ghrelin are transduced by GHSR-1a (18). In somatotroph cells, the activation of GHSR-1a by ghrelin induces GH release through enhanced phospholipase C activity, protein kinase C and intracellular calcium mobilization (19). Attempts to elucidate the peripheral cardiovascular effects of ghrelin have identified several signaling mechanisms involving both classical G-protein effectors and G-protein-independent pathways, highlighting the complexity of GHSR-1a activation (20,21). Ghrelin has been shown to modulate the extracellular signal-regulated kinase (ERK), Akt kinase and cyclic AMP/phorbol myristate acetate pathways (22), and also enhances the migration of glioma cells regulated by the GHSR-1a, AMP-activated protein kinase (AMPK) and NF-κB pathways (23). It has been demonstrated that β-arrestins also function as scaffold molecules for ghrelin-activated signaling networks, such as ERK1/2 and Akt/protein kinase B signaling (24).

In human aortic endothelial cells, ghrelin promotes nitric oxide (NO) synthesis by the GHSR-1a, phosphatidylinositol 3-kinase (PI3K)/Akt and endothelial NO synthase (eNOS) pathways (25). Activation of eNOS and NO production induced by ghrelin depend on a ghrelin receptor/Gq protein/calcium-dependent pathway, leading to stimulation of AMPK and Akt activation (26). In addition, the stimulatory action of ghrelin can be blocked by knockdown of GHSR-1a (27).

## 3. GHSR-1a expression in cardiac remodeling

Remodeled myocardium in adult obese mice over-nourished in early life has been demonstrated to be associated with higher phosphorylation of GHSR-1a, PI3K and AKT, but not with that of AMPK. The increased GHSR-1a content and expression in the heart in obese mice was associated with increased PI3K content, as well as increased AKT content and phosphorylation (28). It has been demonstrated that, through the activation of GHSR-1a, ghrelin produced a positive inotropic effect on ischemic cardiomyocytes and protected them from ischemia/reperfusion injury, likely by regulation of intracellular calcium levels (29).

A GH releasing hormone agonist (GHRH-A) has been demonstrated to markedly improve cardiac function and prevent cardiac remodeling after MI in rats. The effects occurred without increases in the circulating levels of GH and insulin-like growth factor 1 (IGF-1). Therefore, activation of GHRH provides a unique therapeutic approach to reversing remodeling after MI (30,31). These effects were found to be direct and did not involve the GH/IGF-1 axis, as the circulating levels of these hormones were not increased by GHRH-A treatment. GHRH was found to induce dose-dependent calcium mobilization in HEK 293 cells stably transected with GHSR-1a, an effect not observed in wild-type HEK 293 cells (32). This suggests that GHRH interacts with the ghrelin receptor GHSR-1a and modifies the ghrelin-associated intracellular signaling pathway. Ghrelin and GHSR-1a expression have been investigated in different myocardial areas of patients with chronic heart failure (CHF) and compared with that of subjects with healthy hearts (33), to better define the ghrelin signaling in the networks regulating cardiac function and its potential as a target for the diagnosis and treatment of CHF. Ghrelin expression was decreased in the atrium and ventricles of the hearts of patients with CHF, whereas GHSR-1a expression was increased. Ghrelin reduction in CHF hearts may reflect maladaptive processes, whereas GHSR-1a increase may represent a compensatory mechanism.

## 4. Conclusions

Myocardial ischemia and MI lead to contractile dysfunction and remodeling. A major cost for public health is the development of heart failure. One of the most important factors for improving the prognosis after MI is the attenuation of adverse myocardial remodeling and pathological left ventricular hypertrophy. It is well known that several key signaling pathways are involved in the remodeling process, such as the PI3K/Akt (34) and AMPK (35) signaling pathways. Given such complexity in GHSR-1a signaling, we hypothesize that GHSR-1a may play an important regulatory role in cardiac protection. However, to date, there is no direct experimental evidence supporting the correlation between GHSR-1 and cardiac remodeling. Therefore, further studies are required.
